# Characteristics and potential clinical applications of the extracellular vesicles of human pathogenic Fungi

**DOI:** 10.1186/s12866-023-02945-3

**Published:** 2023-08-19

**Authors:** Amir Ullah, Yiyi Huang, Kening Zhao, Yuneng Hua, Shafi Ullah, Mujeeb ur Rahman, Jingyu Wang, Qian Wang, Xiumei Hu, Lei Zheng

**Affiliations:** 1 Department of Laboratory Medicine, Nanfang Hospital, Southern Medical University, Guangzhou, People’s Republic of China; 2 Center for Clinical Laboratory, Zhujiang Hospital, Southern Medical University, Guangzhou, People’s Republic of China; 3 La Trobe Institute for Molecular Science, La Trobe University, Melbourne, Australia; 4 Department of pharmacy, COMSATS University Islamabad, Abbottabad Campus, Abbottabad, Pakistan; 5 Biofuels Institute, School of the Environment and Safety Engineering, Jiangsu University, Zhenjiang, China

**Keywords:** Extracellular vesicles, Fungi, Biogenesis, Composition, Immunomodulation, Biomarkers, Therapeutic applications

## Abstract

Extracellular vesicles (EVs) are a heterogeneous group of lipid membrane-enclosed compartments that contain different biomolecules and are released by almost all living cells, including fungal genera. Fungal EVs contain multiple bioactive components that perform various biological functions, such as stimulation of the host immune system, transport of virulence factors, induction of biofilm formation, and mediation of host–pathogen interactions. In this review, we summarize the current knowledge on EVs of human pathogenic fungi, mainly focusing on their biogenesis, composition, and biological effects. We also discuss the potential markers and therapeutic applications of fungal EVs.

## Introduction

Extracellular vesicles are phospholipid bilayer nanostructures derived from living cells in the extracellular milieu [1]. These membrane particles are secreted by cells of all three domains: prokaryotes, eukaryotes, and archaea [2]. Their diameter ranges from 20 nm to approximately 1 μm. Based on their size, the newest grouping of EVs separates them into two major groups: small EVs or endosomal-origin EVs named “exosomes” (up to 150 nm) and medium/large EVs or microvesicles (> 150 nm) [3]. Although the characteristics and functional roles of EVs have been extensively studied in mammalian systems [4], our understanding of EVs in fungi remains relatively limited. EVs derived from fungi were first identified in *Cryptococcus neoformans* during a study of the trans-cell wall transport mechanisms of capsular polysaccharides [5]. Since then, EVs have been reported in various other virulent and non-virulent fungal species [6]. Similar to the mammalian, bacterial, and plant EVs, fungal EVs also contain proteins, carbohydrates, pigments, nucleic acids, toxins, and other bioactive molecules in their intercellular matrices or on their surface. These cargoes are transported to the extracellular environment for various biological functions [7–14]. Once EVs are released into the extracellular space, they migrate long distances until they encounter another cell or environment to connect with [15]. Several EV components are conserved among various fungal species and strains, while some are species-specific [8, 16]. Fungal EVs with immunogenic properties exert immunotherapeutic effects, and hence, can be used as potent tools for various therapeutic applications [17–19].

In this review, we summarize the biogenesis, composition, biological effects, and potential therapeutic applications of EVs of human pathogenic fungi. We also discuss the roles of EVs of non-pathogenic fungi in some sections for comparison. Finally, we outline the current challenges in fungal EV research.

## Biogenesis of fungal EVs

Most studies on EV biogenesis mechanisms have focused on mammals; however the biogenesis mechanisms of fungal EVs remain unclear [6]. Extreme variations in the morphology, size, cargo, and composition of EVs suggest the presence of different EV biogenesis pathways in cells [1, 20, 21]. Several cytoplasmic proteins without any secretory signals have been discovered in fungal EVs, suggesting that EVs may be derived from cytoplasmic fractions [22]. Multivesicular bodies and membrane budding also play roles in EV biogenesis process [9, 20, 23, 24]. EVs are also produced via an invagination process (inverted macropinocytosis) and the vesicles are subsequently released into the periplasmic vacuum [22]. Moreover, EVs may be derived from vesicle-containing vacuoles that are fused with the plasma membrane subsequently [21].

**Fig. 1:**
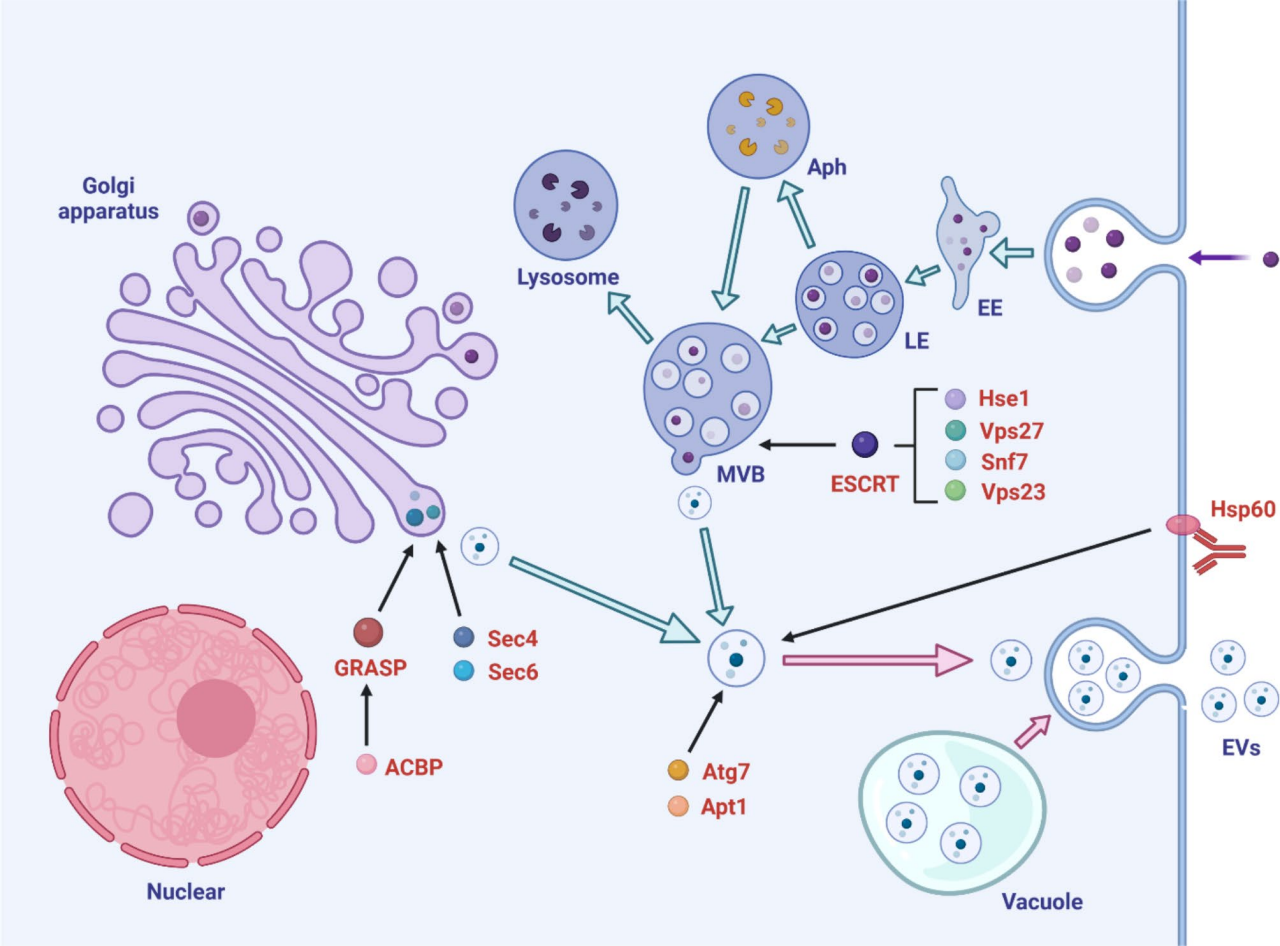
Schematic diagram of fungal extracellular vesicle (EV) biogenesis. Fungal EV biogenesis is regulated by multiple secretory regulators, including Golgi apparatus secretory pathways, ESCRT machinery and other biogenesis regulators. EVs may also be derived from vesicle-containing vacuoles in *C. neoformans* [21]. Mutant cells lacking the Golgi reassembly and stacking protein (GRASP) and autophagy-related protein 7 (Atg7) exhibit altered EV size in *C. neoformans* [26]. Mutation in acyl coenzyme A-binding protein (ACBP) in *D. discoideum* [28] and *S. cerevisiae* [29] indicate a reduction in components of EVs. Mutation in *SEC4* gene alter the production of vesicles in *S. cerevisiae* [20]. *SEC6* gene suppression decreases the secretion of virulence-related molecules into EVs of *C. neoformans* [25]. ESCRT sub-units, Snf7 and Vps23, influence EV proteins in *S. cerevisiae* [20]. ESCRT sub-units, Hse1 and Vps27, influence biofilm EV formation in *C. albicans* [30]. Deletion of *APT1* gene encoding lipid flippase influences the size of EVs and EV-based export of soluble GXM in *C. neoformans* [32, 33]. Attachment of monoclonal antibodies targeting Hsp60 to *H. capsulatum* fungal cells alters the EV cargo and release [43, 44]. EE-early endosome; LE-late endosome; Aph-autophagosome; MVB-multivesicular body

Characterization of EVs formed by mutants with defects in several pathways is the key method used to study vesicle biogenesis in fungi. It’s been demonstrated that both Golgi apparatus secretory pathways and endosomal sorting complex required for transport (ESCRT) machinery are involved in fungal EV biogenesis, as well as a variety of other biogenesis regulators. Figure 1 shows the detailed biogenesis mechanisms of fungal EVs.

### Golgi apparatus secretory pathways

It’s been reported that Golgi apparatus secretory regulators are associated with fungal EVs biogenesis. For instance, in *Saccharomyces cerevisiae*, a mutation in the *SEC4* gene responsible for encoding exocytic Rab GTPase (required for post-Golgi secretory vesicle formation) alters the production of vesicles [20]. In *C. neoformans*, Sec6 is implicated in the exocytosis of post-Golgi secretory vesicles to the plasma membrane. Suppression of *SEC6* using a small interfering RNA decreases the secretion of virulence-related molecules into EVs [25]. Sec1 is associated with the integration of Golgi-derived EVs, and the cell membrane participates in EV formation as well. However, deletion of *SEC1* does not influence the secretion of EVs [20].

Golgi reassembly and stacking protein (GRASP) and autophagy-related protein 7 (Atg7) are related to EV secretion, and mutant cells lacking GRASP and Atg7 exhibit altered EV size [26]. GRASP is involved in EV-mediated mRNA transport and is a significant modulator of virulence in *C. neoformans* [27]. Mutant cells lacking acyl coenzyme A-binding protein (ACBP), a GRASP expression needed for conventional secretion in *Dictyostelium discoideum* [28] and *S. cerevisiae* [29] exhibit a reduction in EV components compared to the wild-type cells [20].

Although alterations in these conventional secretory genes affect EV formation, they do not completely inhibit the development of EVs, suggesting the involvement of other functional pathways in the generation and release of EVs.

### ESCRT machinery

Apart from Golgi apparatus secretory pathways, ESCRT machinery is also demonstrated to be involved in the biogenesis of fungal EVs. ESCRT is associated with the development and functions of multivesicular bodies (MVBs). For example, Snf7 and Vps23 influence EV proteins in *S. cerevisiae* [20]. In *Candida albicans*, biofilm EVs contain ESCRT sub-units, Hse1 and Vps27, and mutations in different ESCRT sub-unit homologs result in reduced EV formation compared to that in wild-type strains [30]. Moreover, EVs from ESCRT-knockout yeast strains exhibit enrichment of cell wall-rebuilding enzymes, particularly chitin synthases Chs1 and Chs3, and the glucan synthase sub-unit, Fks1 [10]. Absence of Vps27 in the ESCRT complex results in the accumulation of MVBs and discharge of larger EVs in *C. neoformans* [31].

### Other biogenesis regulators

In addition to the ESCRT apparatus, several regulators may affect EV production and cargo composition. Deletion of the *APT1* gene encoding lipid flippase alters the size of *C. neoformans* EVs and the EV-based export of soluble glucuronoxylomannan (GXM) [32, 33]. In *Cryptococcus gattii*, removal of *AIM25*, which encodes the enzyme lipid scramblase, results in the formation of larger EVs with altered RNA content [34]. In *Candida albicans*, mutant species deprived of genes encoding phosphatidylserine synthase/decarboxylase (CHO1/(PSD1 and PSD2) influence the morphology, immunogenicity, and composition of EVs, which is highly suggestive of an association between the metabolism of lipids and constituents of EVs [35]. Other regulators also play a role in the release of EVs. For example, deletion of the chitin synthetase (*CHS*) gene in *C. neoformans* inhibits EVs release [36]. High EV production is observed in a *C. neoformans* strain lacking Cln1, a protein associated with cell cycle progression [37]. In addition, the removal of CAP10, a putative xylosyltransferase, considerably reduces the size of EVs [38]. Intracellular vesicular clusters of *S. cerevisiae* promote the production and selection of proteins associated with EV secretion [39]. Moreover, reduced expression of cryptococcal microvesicle marker protein 14-3-3 [40] decreases the GXM and protein content in EVs and decreases acid phosphatase and laccase activities [41].

Furthermore, the environmental stimuli, host-posed conditions and metabolic mechanisms are thought to influence the release of EVs, as well as their size and composition [7, 22, 30, 42–44]. The attachment of protective or non-protective monoclonal antibodies (MAbs) targeting Hsp60 to *Histoplasma capsulatum* fungal cells has been shown to results in altered EV cargo and release [43, 44]. Moreover, treatment with caspofungin, an antifungal drug, enhances the release of EVs from *S. cerevisiae* [10]. Interestingly, EVs release can also be influenced by EV size. Only multiple vesicle-leaving events (in which a group of vesicles is released simultaneously) release EVs larger than 100 nm in *C. neoformans*, whereas smaller vesicles may be released in multiple or single vesicle-leaving events [9]. These studies highlight the complicated mechanisms related to the formation and secretion of EVs (Fig. 1).

### Cell wall-crossing mechanisms of fungal EVs

The intricate and vibrant cell wall surrounding the fungal cells is generally considered the ultimate barrier for the secretion of fungal EVs to reach the external environment [45]. Therefore, the ability of fungal EVs to cross the cell wall is a fundamental requirement. In encapsulated fungal species, EVs must also cross the capsules of fungal cells such as those related to the *Cryptococcus* group [5]. However, the cellular mechanisms underlying the secretion of fungal EVs across the cell wall and capsule have not yet been clearly elucidated. The cell wall is primarily composed of a polysaccharide matrix in which various proteins and lipid groups are incorporated [45]. Three nonexclusive hypotheses have been put forth to explain the cell wall crossing mechanism of fungal EVs. First, protein channels present in the periphery of the cells can guide the fungal EVs to the extracellular space. Second, once fungal EVs are released from the cell membrane, the turgor pressure exerted by the cells forces them through the pores in the cell wall. Finally, hydrolytic enzymes loosen the cell wall by digesting specific areas of the cell wall, which allows the passage of fungal EVs through the cell wall [46–48].

Data supporting these non-mutually exclusive hypotheses regarding EVs passage through the fungal cell wall are dispersed in the literature. The size of the pores in the cell wall differs by strain and may be adjusted under distinct conditions depending on the cell wall remodeling enzymes, culture growth phase, extracellular pH, and deposits, such as melanin. The diameter of the pores present in the cell wall of *S. cerevisiae* may increase to 400 nm in response to stress [49]. This alteration represents a possible channel for larger EVs to cross cell walls. However, decrease in cell wall pore size in cryptococci can contribute to the aggregation of vesicle-like structures “trapped vesicles” seen in the region between cell wall and plasma membrane [9, 50]. Recent research has suggested that fungal cell walls have viscoelastic properties that may allow the transportation of membranous particles. Amphotericin B-containing liposomes with diameters of 60–80 nm penetrated the cell walls of *C. albicans* and *C. neoformans* from the outer environment of the cell and approached the plasma membrane in their original form, although the estimated size of the pores present in the cell wall was notably low (approximately 5.8 nm) to permit their transport [48]. Cryo-SEM analysis showed that single and multiple vesicles interacted directly with the cell wall of *C*. *neoformans* without any apparent trans-cell wall channels or pores, encouraging their passage [9]. This is in contrast to the channels in fungi that steer vesicle secretions.

Cell wall remodeling may be an effective mechanism for vesicle release. Proteomics has shown that several enzymes involved in cell wall remodeling, such as glucanases, are found in EVs, facilitating their passage through the cell wall [10, 51, 52]. Additionally, EVs are produced by polymorphological nuclear granule cells. These EVs are bound to the cell wall of *Aspergillus fumigatus* and are also capable of entering the hyphae of fungal cells, which results in the modification of the morphology of the fungal cell walls [53]. These studies highlight the dynamic and flexible structure of the cell wall along with its viscoelastic characteristics, which provide innovative information about the movement of vesicles through the cell wall to approach the extracellular space [6].

### Composition of fungal EVs

Several studies have shown that fungal EVs carry various bioactive substances. Using EVs as a medium, these substances are involved in multiple biological activities. Fungal EVs mainly contain the following substances:

#### Proteins

Fungal EVs cargo is composed of a combination of different proteins that may or may not contain secretory signals. Fungal EVs contain proteins with biological roles in the biogenesis of cell wall architecture, cell membrane, response to stress, virulence/pathogenesis, cell signaling, sugar metabolism, lipid metabolism, cell growth/division, and transportation. Most of these proteins are shared among EVs from different fungal species, including *C. neoformans*, *C. albicans*, *Paracoccidioides braziliensis*, *H. capsulatum*, and *S. cerevisiae*, and species-specific proteins have also been widely studied [8, 20, 54–56].

In *C. neoformans*, two previous proteomic analyses reported 92 and 202 proteins in the EVs extracts [9, 21]. Enzymes involved in capsule biosynthesis, such as UDP-glucose dehydrogenase and UDP-glucuronic acid decarboxylase, are specifically found in *C. neoformans* EVs [21]. Recently, the analysis of EVs protein cargo from three cryptococcal species revealed many membrane-bound protein families, including Tsh proteins bearing the SUR7/PalI motif. Proteins involved in various biological processes have been identified in the EVs cargo, including Mp88 and components of the Cda, Gox, and Ril protein families [57]. In a previous study, the ability of *C. gattii* EVs to trigger an increased intracellular proliferation rate was eliminated when pretreated with proteinase K, indicating that *C. gattii* EVs contain proteins related to pathogenicity [15]. EVs of *H. capsulatum* revealed the presence of proteins related to REDOX, including superoxide dismutase, thiol-specific antioxidants, and catalase B, which are involved in fungal defense. Various enzymes, such as glucanase and endochitinase, are also involved in the hydrolysis of cell wall constituents that facilitate the secretion of EVs. Proteins involved in the transport and fusion of vesicles, particularly those of the Rab family, have also been identified [55]. Proteomic analysis of *P. braziliensis* EVs revealed a varied and complex distribution of proteins with various biological functions [8]. Protein content was compared between the EVs of *P. braziliensis* and those of *C. neoformans*, *S. cerevisiae*, and *H. capsulatum*. Twenty-six proteins were found to be common among these four fungal species. Additionally, 72 proteins are common to *P. braziliensis* and at least two other species [8]. Proteomic analysis of EVs derived from *A. fumigatus* protoplasts revealed the presence of proteins related to lipid and sugar metabolism, cell wall biosynthesis, and pathogenic processes. In EVs from *A. fumigatus* protoplasts, proteins not expected to be present in the extracellular environment have been identified in EVs from *A. fumigatus* protoplasts [23]. This finding is in agreement with various studies on the protein composition of fungal EVs [21, 51, 54, 55].

In *C. albicans*, the first proteomic study of EVs reported 75 proteins that fell into nine functional classes, most of which were related to the cell wall [58]. A label-free quantitative assessment identified 47 proteins that were enriched in *C. albicans* EVs compared to those in planktonic cells. These proteins were refined to 22 possible eV protein markers in *C. albicans*, including claudin-like Sur7 family proteins (Sur7 and Evp1). Additionally, azole-resistant proteins, including Cdr1 and Cdr2, have been detected [59]. Another proteomic study comparing planktonic cells and biofilm EVs in *C. albicans* showed that 34% of the protein cargo was specific to biofilm EVs [30]. Analysis of EVs from non-*albicans Candida* species (NAC) revealed the presence of 42 extracellular and surface-connected proteins from *Candida glabrata*, 33 from *Candida parapsilosis*, and 34 from *Candida tropicalis* [60]. Of the EVs proteins identified in *Candida* species, many were also found in EVs from other fungi, including *S. cerevisiae* and other species that are pathogenic to humans [61]. Some proteins present in fungal EVs are assumed to be moonlighting proteins [60].

A quantitative large-scale proteomic study comparing the protein content of EVs and whole-cell lysates of *Malassezia sympodialis* revealed the presence of many proteins that were enriched in EVs, including two allergens (Mala s1 and Mala s7) and catalytic enzymes (helicases, ligase, histone, tRNA synthetase, ribonuclease, and deacetylase) [62]. In *Alternaria infectoria*, a phytopathogen as well as an opportunistic pathogen in humans, proteomic analysis of EVs cargo revealed the existence of proteins related to cell metabolism and pathogenicity. Of the class of carbohydrate metabolism proteins, the beta-xylosidase, a fungi allergen, was identified in *A. infectoria* EVs [63]. EVs of *S. cerevisiae* contain proteins related to cell metabolism, cell wall formation, and stress responses [10, 20, 39, 64–67]. The EVs protein cargo of *S. cerevisiae* was parallel to that of *C. neoformans* and *H. capsulatum* regarding protein classification [21, 55], suggesting that EVs composition is maintained in pathogenic and non-pathogenic fungi. *S. cerevisiae* is an important model system for analyzing EV-mediated prion transport. A fungal prion, Sup35p, is exported via EVs in soluble and infectious forms [68], and EVs can pass prions to recipient cells where protein accumulation occurs [69].

Proteomic approaches have identified proteins enriched in EVs from many fungal species involved in host–pathogen interactions [8, 20, 21, 54, 55, 60]. In addition, proteins responsible for transport across membranes, such as ATP and ADP carriers and ATPase, have also been demonstrated in EVs of *P. braziliensis*, *S. cerevisiae*, *C. neoformans*, and *H. capsulatum* [8, 20, 21, 55]. Cell wall remodeling enzymes, including Scw4 and Exg1, or proteins associated with the cell wall, such as Ecm33, have also been detected in the EVs of *S. cerevisiae* [20]. This indicated a common phenomenon involving the presence of these proteins in EVs among different fungal organisms, which may be correlated with their secretion. The proteins enriched in fungal EVs and their functional classifications are listed in Table 1.

**Table 1:** Functional classification of proteins enriched in fungal extracellular vesicles (EVs)

	*C. neoformans*	*C. gattii*	*C. albicans*	NAC	*P. braziliensis*	*A. fumigatus*	*H. capsulatum*	*T. marneffei*	*A. infectoria*	*S. cerevisiae*
Cell metabolism	√		√	√	√	√	√	√	√	√
Cell wall formation	√		√	√		√	√			√
Signal transduction	√			√	√		√	√		
Stress response			√	√	√	√	√	√		√
Transcription and translation				√	√					√
Enzymes	√		√	√	√	√	√	√		
Pathogenicity	√	√	√			√		√	√	
Heat shock proteins	√							√	√	√
Immunogenic proteins	√								√	
Biofilm biogenesis			√							
References	[9, 21, 57]	[15]	[30, 35, 54, 58, 59, 70–72]	[60, 70]	[8]	[73, 74]	[44, 55, 75, 76]	[77]	[63]	[10, 20, 39, 64–67]

NAC- non-*albicans Candida* species

#### Lipids

Fungal lipids play significant roles in biofilm formation, drug resistance, microdomain production, and EV production. Any alteration in the composition of lipids in the fungal membrane and EVs alters fungal pathogenicity [78]. Lipid composition of fungal EVs is suggested to be similar to that of cells of the same origin. However, advanced investigations have revealed that only a few lipids are designated as a particular type of EVs [79]. The lipid composition of EVs from pathogenic fungal species mainly includes sterol derivatives, phospholipids, and glycosphingolipid GlcCer, which are essential constituents of the plasma membrane [5]. Phospholipids, such as phosphatidylcholine, phosphatidylethanolamine, and phosphatidylserine, are the major components of EVs membranes across a range of fungal species, including *C. neoformans*, *H. capsulatum*, and *P. braziliensis* [5, 16, 55, 80].

EVs derived from *C. neoformans* are composed of phospholipids, GlcCer, and sterols, such as ergosterols and obtusifoliol [5, 80], similar to the lipids reported for *C. albicans* EVs [35, 54]. GlcCer and sterols are the main neutral lipids present in *C. albicans* EVs. GlcCer is critical for dimorphism and disease progression in *C. albicans* [81]. Lipidomic analysis of EVs from *C. albicans* and *Candida auris* demonstrated higher ergosterol concentrations in the EVs of *C. albicans* than in those of *C. auris* [54]. Furthermore, the concentrations of diacylglycerols and triacylglycerols are increased in *C. albicans* [35]. In contrast, most glycerophospholipids were dominant in *C. auris* [70]. Sphingolipids, such as hexosylceramides, were also found in both species. EVs derived from biofilm-forming *C. albicans* possess phospholipids, such as phosphatidylethanolamine, phosphatidylinositol, and phosphatidylcholine, together with sphingolipids [30]. In EVs of other *Candida* species, including *C. glabrata*, *C. parapsilosis*, and *C. tropicalis*, lipid analysis demonstrated the presence of lecithin phospholipids in all three species, with *C. parapsilosis* exhibiting the highest quantity of lecithin compared to the other two species [82].

In *H. capsulatum* EVs, different lipids, particularly phosphatidylcholine, phosphatidylserine, and phosphatidylethanolamine, have been identified at specific concentrations in H. capsulatum EVs. All these lipids are considered to serve multiple purposes and are the main components of the structure, along with the biological functioning of the double-layered lipids in both mammals and fungi [55]. *H. capsulatum* EVs contain ergosterols, diacylglycerides, triacylglycerides, sphingomyelins, phosphoinositol ceramides and lysophospholipids [76]. Lipidomics comparison of fatty acids and sterols between whole-cell lipid extracts and EVs extracts from two separate isolates of *P. braziliensis* showed compositional similarities and proportional differences. The sterol composition of EVs was brassicasterol, followed by ergosterol and lanosterol, which were prevalent in both isolates. In addition, *P. braziliensis* EVs are rich in phospholipids, including phosphatidylethanolamine, phosphatidylserine, phosphatidylglycerol, phosphatidylcholine, phosphatidic acid, and phosphatidylinositol [16]. Table 2 lists the types of lipids enriched in the EVs from different fungi.

**Table 2:** Lipids enriched in fungal EVs

	*C. neoformans*	*C. albicans*	NAC	*P. braziliensis*	*H. capsulatum*
Phospholipids	√	√	√	√	√
Sterols	√	√	√	√	√
Glycerophospholipids		√	√		
Lysophospholipids		√	√		
Sphingomyelins		√	√		√
Ceramides	√			√	√
Fatty acids			√	√	
Glycosphingolipids		√			
Glycerides					√
References	[5]	[30, 35, 54, 70]	[60, 70]	[16]	[55, 76]

NAC- non-*albicans Candida* species

#### Carbohydrates

Complex carbohydrates, such as GXM have been shown to be exported by fungal EVs [56] across the cell wall [5]. GXM is a unique feature of *C. neoformans* and is an essential virulence factor [83]. GXM has been found to be enriched in EVs of *Cryptococcus* species, both in *C. neoformans* and *C. gattii* [5, 34].

In *A. fumigatus*, carbohydrate analysis showed the presence of mannosyl (Man) and galactosyl (Gal) and a significant amount of glucosyl (Glc) and N-acetyl-galactosaminyl (GalNAc) residues in EVs derived from regenerating protoplasts [23]. GalNAc is an epitope of galactosaminogalactan (GAG), which is a part of the extracellular matrix of *A. fumigatus* and is associated with virulence [84, 85]. In *P. braziliensis*, EVs carbohydrates are comprised of glucose, mannose, and galactose residues containing a heavy molecular mass *α*-4,6-glucan and galactofuranosylmannan, which is probably an oligomer having a 2-*α*-Manp backbone chain linked to *β*-Galf (1,3) and *α*-Manp (1,6) residues [86]. EVs from *P. braziliensis* carry highly immunogenic *α*-linked galactopyranosyl epitopes present both on the surface and inside the EVs [87].

In addition, lectin microarrays have shown the presence of terminal Man and GlcNAc residues exposed on the surface of *P. braziliensis* and *Paracoccidioides lutzii* EVs identified by DC-SIGN receptors [86]. EVs derived from *C. albicans* biofilms displayed a high degree of consistency in composition with the matrix material, including proteins and polysaccharides, primarily glucan and mannan [30]. These findings reveal that EVs may be a significant source of matrix material and contribute to resistance against antifungal agents. A description of the carbohydrates enriched in the fungal EVs is presented in Table 3.

**Table 3:** Carbohydrates enriched in fungal EVs

	*C. neoformans*	*C. gattii*	*C. albicans*	*P. braziliensis*	*P. lutzii*	*A. fumigatus*
Glucuronoxylomannan	√	√				
Mannan-Glucan			√	√	√	
N-acetyl-galactosaminyl				√	√	√
Mannosyl				√	√	√
Galactosyl				√	√	√
Glucosyl				√	√	√
Lectins				√	√	
Surface carbohydrates				√	√	
α-linked galactopyranosyl				√		
References	[5, 34]	[15, 34]	[30]	[87, 88]	[88]	[73]

#### Nucleic acids

EVs cargo is generally composed of diverse genetic materials. EVs allow the flow of genetic information owing to the data contained in their RNA and DNA cargoes. However, mitochondrial and genomic DNA are rarely found in EVs [89–91]. However, most EVs are rich in RNA, including small non-coding RNAs (such as microRNAs [miRNAs], small nuclear RNA [snRNAs], small nucleolar RNAs [snoRNAs], and tRNA fragments [tRFs]), long non-coding RNAs, and mRNAs that have been identified in EVs from both eukaryotic and prokaryotic organisms [92]. RNA composition in fungal EVs has been described in a range of species including *C. neoformans, P. braziliensis*, *C. albicans*, *S. cerevisiae*, *C. gattii*, *Pichia fermentans*, *M. sympodialis*, and *H. capsulatum* [13–15, 93, 94].

The earliest detailed study on the RNA content in fungal vesicles, known as evRNAs, characterized C.*albicans*, *P. braziliensis*, *S. cerevisiae*, and *C. neoformans* [14]. In these organisms, EVs transport small RNA molecules secured by EVs membranes through the degradation of exogenous rNase. A previous fungal EVs study showed that most EVs small RNA molecules are less than 250 nucleotides in length on average. Overall, 1246 conserved miRNA-like sequences (milRNAs) were identified, of which 20 sequences were common among the four tested species (*P. braziliensis*, *C. neoformans*, *C. albicans*, and *S. cerevisiae*). Interestingly, among the ncRNA species, snoRNAs and tRFs were the most frequently observed EVs RNAs in all tested species [14]. Comparative analysis of EVs RNA content derived from the two strains of *H. capsulatum* revealed a total of 124 mRNAs with significant differences in their composition. Strain-specific short reads ranging from 25 to 40 nucleotides were also identified. Half of these fragments were associated with the reverse transcript strand, indicating that milRNA was present in the fungal EVs. Because these two highly virulent *H. capsulatum* strains produce EVs that are abundant in RNA classes related to stress responses and translation, an association between EVs RNA and virulence has also been proposed [12]. Comparison of sub-populations of RNA (less than or more than 200 nucleotides in length) in EVs from *P. braziliensis* (Pb18 and Pb3) and *P. lutzii* (Pb01) demonstrated that 15 ncRNAs were shared among all samples. snoRNAs were enriched in *P. braziliensis* EVs, whereas the proportions of snoRNAs, tRNA, and rRNA in *P. lutzii* EVs were identical. Putative exonic small RNAs are abundant in EVs of Pb18 strain [95]. sRNA classes are involved in modulating transcription and translation. These studies showed that variations in virulence among fungal isolates could be attributed to their distinct EVs RNA contents [95].

Analysis of EVs produced by yeast and pseudohyphal forms of *Pichia fermentans* showed higher RNA content in EVs from the yeast form. In pseudohyphal EVs, stress-induced spliceosomes and miRNAs associated with hypoxia and cell differentiation are highly expressed. Nevertheless, miRNAs or snRNAs involved in splicing regulation or RNA degradation in both growth forms were similarly expressed in EVs [93]. EVs derived from the human fungal pathogen *Rhizopus delemar* contain many RNA species, such as mRNAs, lncRNAs, tRNAs, and miRNAs [96]. Next-generation sequencing was performed to investigate the RNA profiles of EVs derived from *C. auris*. The results showed that EVs contain 104 sequences of non-coding RNAs (tRNAs and tRNAs-half) and 563 sequences of messenger RNAs, snoRNAs, and rRNAs [70]. *M. sympodialis* also possesses small RNAs which are a collection of 16–22 nucleotides with well-defined start and stop loci. Although no genes encoding the components of the RNAi machinery are found in this organism, they possess an RNAi-independent mechanism for the biogenesis of these small RNA molecules [97].

RNA molecules are considered as crucial players in intercellular communication. Their involvement in communication derives from their ability to control gene expression in recipient cells by sending specific RNAs [98]. EVs have been used for RNA transfer in *C. gattii*. Macrophages infected with a non-outbreak *C. gattii* strain take up EVs originating from a highly virulent *C. gattii* strain and lead to increased survival of *C. gattii* inside macrophages [15]. This indicates that EV RNA is necessary for virulence transmission.

### Effects of EVs on fungal pathogenicity

Fungal EVs contain various bioactive substances. This suggests that they potentially participate in intercellular communication and influence fungal pathogenicity.

#### Biofilm generation

Biofilms are fixed communities of microorganisms that allow cells to firmly adhere to each other and other surfaces and are guarded by a polymeric extracellular matrix composed of polysaccharides [99]. Cells in biofilms exhibit enhanced resistance along with distinctive phenotypes compared to planktonic or unadhered cells, and are also associated with the perseverance of infections [100]. Production of biofilms is a major phenomenon in the pathogenesis of *C. albicans* [101], and the biofilm matrix forms a defense mechanism against antifungal drugs [102]. The cargo of biofilm EVs consists of proteins and carbohydrates, mainly mannan and glucan, similar to those in the biofilm matrix, making EVs a notable factor in establishing the biofilm matrix [30]. As determined by 1 H and 2D 1 H-13 C NMR, the major mannan constituents in the vesicle cargo exhibited structural resemblance to the biofilm matrix mannan–glucan complex, which is a biofilm-related drug resistance-determining factor [103]. The activity of biofilm EVs was analyzed using *C. albicans* ESCRT mutants, which led to decreased biofilm EVs production, decreased matrix polysaccharide levels, and higher fluconazole vulnerability than wild-type *C. albicans*. The “add-back” of EVs from wildtype biofilms reestablishes matrix accumulation and biofilm drug resistance [30]. This finding suggests that the biofilm EVs of *C. albicans* play critical roles in the development of matrices and antimicrobial resistance. Furthermore, biofilms may mediate drug resistance through the overexpression of drug efflux pump proteins [102, 104]. The presence of drug efflux pump proteins in the EVs of *C. albicans* is indicative of the biological function of EVs in azole resistance by transporting efflux pump proteins to non-resistant strains [59]. Additionally, EVs of biofilms have large amounts of oxidative, heat stress, and virulence-inducing proteins, indicating a possible role for EVs in pathogenesis [59].

#### Mediation of fungal virulence

The importance of fungal EVs in mediating virulence has been previously proposed. The fatal human pathogen *C. gattii* secretes EVs to mediate the virulence of neighboring fungal cells [15]. The outbreak lineage of *C. gattii* has the ability proliferates rapidly within host phagocytes [105]. Previously, reports has demonstrated that this rapid proliferation is driven by a ‘division of labor’ mechanism [106]. Division of labor is mediated by the secretion of fungal EVs. Virulent-strain-derived EVs are taken up by infected host macrophages and trigger rapid intracellular proliferation of the non-outbreak lineage [15].

### Effects of fungal EVs on host–pathogen interactions

**Fig. 2:**
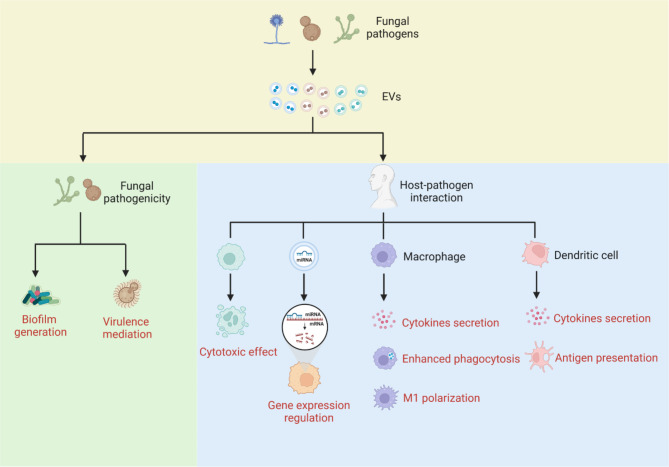
Biological effects of fungal EVs on fungal pathogenicity and host–pathogen interactions. As an important medium of intercellular communication, fungal EVs can mediate both fungal cell–cell communication and host–pathogen interactions. Biofilm EVs carrying biological molecules are involved in biofilm generation and resistance in *C. albicans* [30]. *C. gattii* EVs mediate the virulence of neighboring fungal cells [15]. *C. neoformans* EVs contain glucuronoxylomannan (GXM) and exert both immunosuppressive and cytotoxic effects on immune cells [107–110]. MicroRNAs are significant constituents of EVs in *C. albicans* and *C. neoformans* and modulate the gene expression in host cells [113]. EVs modulate the phagocytosis index and induce cytokine production by macrophages in *C. neoformans* [117]. In *A. flavus*, EVs induce M1 polarization of macrophages [17]. *C. albicans* EVs activate dendritic cells (DCs) for antigen presentation and cytokine secretion [54]

The presence of different bioactive components in fungal EVs increases the probability of their association with prospective hosts. The interaction between fungal EVs and the host mainly occurs through direct biological effects and immunomodulation. Figure 2 provides an overview of fungal EVs in host–pathogen interactions.

#### Direct biological effects on the host

Several studies have demonstrated that virulence-related molecules are exported directly or via fungal EVs, which could have direct biological effects on the host. The virulence factors enriched in fungal EVs and their biological effects on the host are summarized in Table 4.

In *C. neoformans*, GXM is a vital element of the fungal polysaccharide capsule that exerts both immunosuppressive and cytotoxic effects on immune cells [107–110], in order to avoid the killing of fungi by macrophages through phagocytosis [111]. Melanin production enhances the resistance to macrophage phagocytosis and reactive oxygen species derived from host cells [7, 112]. EVs released by *C. neoformans* are also capable of enhancing brain infection, as they boost the transmigration of yeast across the blood-brain barrier [40]. Small RNAs, including microRNAs (miRNAs), are significant constituents of EVs in *C. albicans* as well as *C. neoformans*. The RNAi machinery present in both fungal species suggests the role played by EV-derived small RNA molecules during fungal infections by mimicking endogenous miRNAs to modulate gene expression in host cells [113]. Recently, proteomic analysis demonstrated that EVs derived from *Talaromyces marneffei* contain heat shock proteins and mannoprotein 1, which maintain homeostasis and pathogenicity [77].

Fungal EVs contain components with both pathogenic and immunogenic properties that allow fungal EVs to trigger the host immune response [58]. Phospholipid phosphatidylserine is an essential element of *C. albicans* EVs to activate immune cells, as phosphatidylserine synthase CHO1 deficient EVs are unable to stimulate NF-κB in bone marrow-derived macrophages (BMDMs) [35]. Glucosylceramide is another vital antigenic molecule that triggers immune responses and is found in the EVs of *C. albicans* and *C. neoformans* [114, 115]. EVs of *P. braziliensis* carry epitopes of α-galactopyranosyl, an extremely immunogenic particle, which were recognized efficiently by anti-α-gal antibodies within patients with paracoccidioidomycosis [87]. Recently, proteomic analysis of EVs derived from NAC, such as *C. tropicalis*, *C. parapsilosis*, and *C. glabrata*, demonstrated abundant moonlighting proteins or their orthologs with the ability to induce immune responses, adhesive roles, and pathogenic potential [60].

**Table 4:** Virulence factors enriched in fungal EVs and their direct biological effects on the host

EV source	Virulence factors	Biological effects on the host	References
*C. neoformans*	GXM	Cytotoxic effect, immunosuppression	[7, 107–110, 112–114]
	Melanin	Phagocytosis suppression	
	Glucosylceramide	Immune activation	
	Small RNA	RNAi machinery	
*C. albicans*	Phosphatidylserine, Glucosylceramide	Immune activation	[35, 115]
	Small RNA	RNAi machinery	
NAC	Moonlighting proteins	Immune activation	[60]
*P. braziliensis*	α-Galactopyranosyl	Immune activation	[87]
*T. marneffei*	Heat shock proteins	Homeostasis regulation	[77]
	Mannoprotein 1	Pathogenicity	

NAC- non-*albicans Candida* species

#### Modulation of the host immune response

Recent studies have shown that fungal EVs strongly influence host immunity [17, 116]. In *C. neoformans*, EVs are involved in modulating the phagocytosis index and antimicrobial potential of macrophages, as they induce the secretion of high concentrations of cytokines, such as tumor necrosis factor-alpha (TNF-α), interleukin (IL)-10, transforming growth factor-beta (TGF-β), and nitric oxide (NO). Interestingly, both TNF and NO production, together with an improved capacity to phagocytose and destroy fungal cells, indicate the dual role of EVs in triggering positive and negative activation of macrophages [117]. As observed with *C. neoformans* EVs, immunomodulation has also been reported for *C. albicans* in host immune cells. EVs internalization by dendritic cells (DCs) and macrophages induces the production of IL-12, TNF-α, IL-10, TGF-α, and nitric oxide as well as increased levels of MHC-II and CD86 expression in DCs [54]. This cytokine signature is balanced because anti-inflammatory cytokines (IL-10 and TGF-β) serve as host response breaks that can inhibit or decrease tissue damage induced by an intensified inflammatory reaction.

EVs derived from *M. sympodialis* were found to interact with human keratinocytes and induce increased expression of intercellular adhesion molecule-1, which is indicative of a significant step in the skin defense against this fungal pathogen [118]. EVs from the saprophytic fungus *Aspergillus flavus* stimulate macrophages to produce inflammatory mediators, including TNF-α, IL-1, IL-6, and NO, and induce M1 polarization [17]. Similar results were delineated by EVs derived from *P. braziliensis*, while secondary stimulation promoted the secretion of pro-inflammatory cytokines, such as TNF-α, IL-6, and IL-12, and favored M1 polarization [119].

Macrophages expressing the M1 phenotype play an important role in eliminating fungal pathogens. The development of M1 and inhibition of M2 phenotypic expression have also been demonstrated, as they provide protection against infections caused by *C. neoformans, H. capsulatum*, and *A. fumigatus* [120–122]. EVs from *H. capsulatum* lower the phagocytic activity and intracellular killing by BMDMs [44]. These results indicate the immunomodulatory activity of EVs. Depending on the pathogen, fungal EVs can intensify or attenuate the progression of the infection [110].

### Potential fungal EV markers

Suitable fungal EV markers are required to improve EVs isolation methods and conduct detailed research on the composition and biological functions of EVs. Exosomes from mammals have been purified and analyzed using marker proteins, including CD63, Alix, and TSG101 [123, 124], whereas for all EVs, the markers CD9 and CD81 have been used [125]. However, most of the markers for mammalian EVs do not possess orthologs in fungal EVs [126]. Vps27 and Hse1, proteins of the ESCRT complex, have been found in EVs of *C. albicans* but at very low concentrations [30]. Comparative proteomic analysis of EVs derived from *H. capsulatum, S. cerevisiae*, *P. braziliensis*, and *C. neoformans* has revealed 26 protein orthologs in EVs from all four species that may serve as marker candidates [8]. Nonetheless, these results are less convincing when analyzed for individual fungal species [126]. Hsp70, a marker protein found in mammalian exosomes [127], has also been detected in *C. albicans* EVs. An Hsp71-like protein found in *C. neoformans*, which shares more than 80% sequence similarity with the Hsp70 protein found in the proteomic dataset of *C. albicans* EVs, suggests that Hsp70 could be a possible marker for fungal EVs [126].

A recent analysis revealed 22 possible protein markers, including the claudin-like Sur7 family proteins Sur7 and Evp1, in the EVs of *C. albicans*. Based on their possible topological resemblance to tetraspanin markers used for mammalian EVs, Sur7 and Evp1 have been proposed as putative positive markers for *C. albicans* EVs [59]. Sur7 proteins have also been recently described in EVs from *Cryptococcus* [57]. In another study, a monoclonal antibody (mAb476) that specifically recognized Galactofuranose(Galf)-bearing glycoconjugates of Aspergillus was described. EVs fractions of both Aspergillus culture supernatant and clinical samples of patients with invasive aspergillosis (IA) were recognized by mAb476. This finding suggesting that Galf is considered a potential marker of Aspergillus EVs and Galf-bearing Aspergillus EVs may be used for clinical diagnostic applications of IA [128]. Moreover, the major lipid component GlcCer is an intriguing fungal EV marker, although certain species, including *C. glabrata* and *S. cerevisiae*, do not generate or synthesize this GSL [129]. No other fungus-specific markers have been identified to date. Unstandardized methods for isolating fungal EVs have significantly limited the development of fungal EV markers.

### Potential therapeutic applications of fungal EVs

**Fig. 3:**
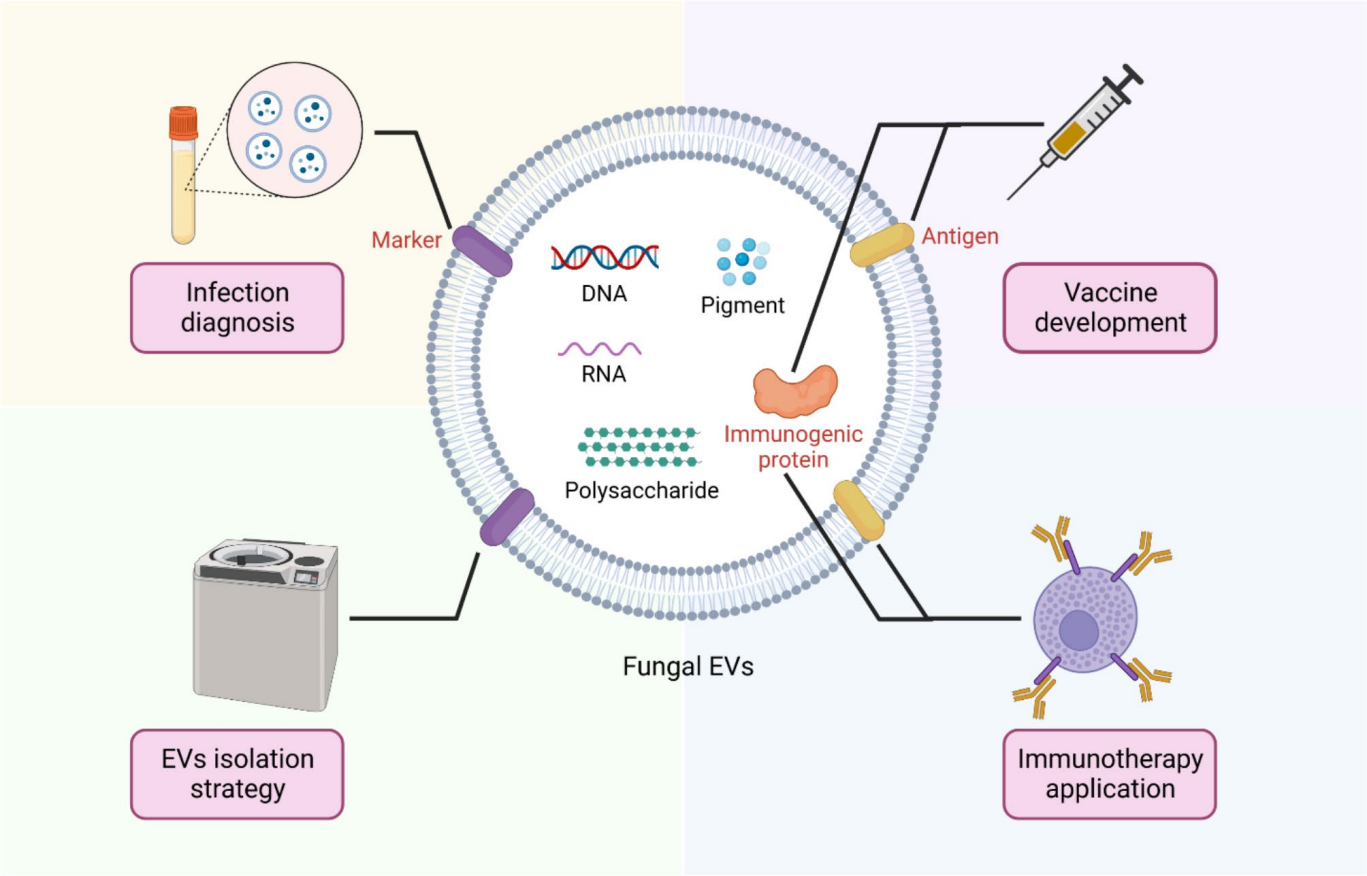
Potential clinical applications of fungal EVs. The membranous structure of fungal EVs allows them to carry various bioactive components, providing fungal EVs great potential for clinical applications. Suitable fungal EVs markers may be useful for fungal infection diagnosis [145] and new EVs isolation strategy [59]. Antigens and immunogenic proteins presented in fungal EVs are able to activate immune response in the host, providing the possibility of fungal EVs in vaccine development and immunotherapy application [54]

There is an increasing need for the development of innovative methodologies for preventing and treating infections caused by pathogenic fungi, as demonstrated by the elevated number of fatalities caused by invasive mycoses. Several attempts have been made to develop vaccines for the prevention of fungal infections, but none have been licensed for public use [110, 130]. In mammalian studies, exosomes are used as biomarkers, cell-free therapeutic agents and cancer vaccine [131, 132]. Fungal EVs have nano-sized structures, contain several immunogenic molecules, and interact with the host in multiple ways, making them suitable candidates for therapeutic applications. Fig. 3 summarizes the potential clinical applications of fungal EVs.

#### Vaccine development

EVs from fungal species possess molecules capable of stimulating immunogenic reactions, endowing fungal EVs with potential for vaccine development. Bgl2 or 1,3-glucosyltransferase is a protein component of EVs in *C. albicans*, which is responsible for the biosynthesis of fungal cell walls and virulence, and can elicit humoral immunity by reacting with the sera of patients with candidiasis [133]. A mouse model showed increased survival following treatment with BgI2 in the vaccine before infection with *C. albicans* [58]. Moreover, the ortholog of the enolase protein (Eno1) present in the EVs of *C. albicans* was found to be abundant as an immunodominant antigenic protein in candidiasis patients [134]. Furthermore, glycoprotein 43 (gp43) is a protein conjugate present in the fungal species *P. braziliensis* that is also present in EVs of *P. braziliensis* [8]. Immunization with P10 lowered the fungal load and improved the survival of healthy or immunosuppressed mice through a process based on the Th1 immune response after lethal challenge with *P. braziliensis* [135–137].

Cryo-EM analysis revealed a novel antigenic structure present on the surface of *Cryptococcus* EVs [138], resembling the spike complexes present on the envelope of viruses [139, 140], suggesting their role as potential vaccines. Indeed, mice immunized with EVs acquired from a mutated capsular strain of *C. neoformans* showed a robust antibody response and substantially extended survival following infection with *C. neoformans* [138]. *Cryptococcal* infection can be prevented through prophylactic immunization, and several antigens of *cryptococcus* have been examined for their potential as vaccines [141, 142]. A mutant strain of *C. neoformans* lacking sterylglucosidase 1 (Sgl1), an enzyme required for sterylglucoside degradation, is nonpathogenic in *cryptococcosis* mouse models. Interestingly, this mutant strain acted as a vaccine that triggered a protective immune response in immunocompromised (CD4 cell-depleted) mice against *cryptococcosis* after challenge with WT *C. neoformans* or *C. gattii*. The deposition of SGs in Δsgl1 mutant may be responsible for this protective immunity. These results are especially relevant in the sense of HIV/AIDS immune deficiency, indicating that a new vaccine strategy against *cryptococcosis* could be given by the Δsgl1 strain [143].

#### Immunotherapy application

Numerous studies have demonstrated the immunotherapeutic potential of fungal EVs [17–19]. Prophylaxis of *Galleria mellonella* with *C. albicans* EVs prior to infection with live *C. albicans* reduces fungal stress and enhances larval survival [54]. Pretreatment of *G. mellonella* with *A. flavus* EVs also decreases CFU levels and enhances the larval survival [17]. Similarly, GXM-containing EVs of *C. neoformans* induce immunity in *G. mellonella* against lethal challenge with *C. neoformans* [144]. EVs from *C. albicans* may exert a protective effect against murine candidiasis [19].

Collectively, these studies demonstrate the potential of fungal EVs to modulate the natural immune response and intercept pathogenesis in vivo to control fungal infection, suggesting native or engineered EVs as promising candidates for therapeutic applications.

## Conclusion and future challenges

In recent years, fungal EVs have gained increasing attention in infectious disease and fungal pathogen research. More extensive and in-depth research will further enhance our understanding of fungal EVs. Despite the recent advancements in fungal EV research, several challenges must be addressed in future studies.

1. Unlike mammalian EVs, the absence of specific biomarkers in fungal EVs has hampered their in-depth study. Detection of new EV markers is a useful resource for studying the biogenesis, cargo packing, isolation, and characterization of fungal EVs and determining their role in pathogenesis.
2. Molecular processes governing the biogenesis, cell wall-crossing mechanisms, and compositional diversity of EVs must be investigated to better understand their biological roles.
3. In-depth studies of the host–pathogen interactions mediated by fungal EVs will provide insights on the pathogenic mechanism of fungi and aid in the development of new treatment strategies for fungal infections.
4. Influence of fungal EVs on recipient fungi and other microorganisms also requires further investigation.
5. Lastly, the potential of fungal EVs as biomarkers for the early diagnosis and therapeutic monitoring of fungal infections needs to be assessed in future studies.

## Data Availability

Not applicable.
